# Impact of monthly headache days on anxiety, depression and disability in migraine patients: results from the Spanish Atlas

**DOI:** 10.1038/s41598-021-87352-2

**Published:** 2021-04-15

**Authors:** P. Irimia, M. Garrido-Cumbrera, S. Santos-Lasaosa, M. Aguirre-Vazquez, J. Correa-Fernández, I. Colomina, P. Pozo-Rosich

**Affiliations:** 1 Department of Neurology, Clínica Universidad de Navarra, Av. Pío XII 36, 31008 Pamplona, Spain; 2 Navarra’s Health Research Institute (IDISNA), Pamplona, Spain; 3 Headache Study Group of the Spanish Neurological Society (GECSEN), Pamplona, Spain; 4 Health and Territory Research (HTR), Universidad de Sevilla, Sevilla, Spain; 5 Department of Neurology, Hospital Clínico Universitario Lozano Blesa, Zaragoza, Spain; 6 Instituto de Investigación Sanitaria IIS Aragón, Zaragoza, Spain; 7 Novartis Farmacéutica Spain, Barcelona, Spain; 8 Headache Unit, Neurology Department, Vall d’Hebron University Hospital, Barcelona, Spain; 9 Spanish Patient’s Association of Migraine and Headache (AEMICE), Madrid, Spain; 10 Headache Research Group, VHIR, Universitat Autónoma de Barcelona, Barcelona, Spain

**Keywords:** Neuroscience, Psychology

## Abstract

Identifying highly disabled patients or at high risk of psychiatric comorbidity is crucial for migraine management. The burden of migraine increases with headache frequency, but the number of headache days (HDs) per month after which disability becomes severe or the risk of anxiety and depression is higher has not been established. Here, we estimate the number of HDs per month after which migraine is associated with higher risk of anxiety and depression, severe disability and lower quality of life. We analysed 468 migraine patients (mean age 36.8 ± 10.7; 90.2% female), of whom 38.5% had ≥ 15 HDs per month. Our results show a positive linear correlation between the number of HDs per month and the risk of anxiety (r = 0.273; p < 0.001), depression (r = 0.337; p < 0.001) and severe disability (r = 0.519; p < 0.001). The risk of anxiety is higher in patients having ≥ 3HDs per month, and those with ≥ 19HDs per month are at risk of depression. Moreover, patients suffering ≥ 10HDs per month have very severe disability. Our results suggest that migraine patients with ≥ 10HDs per month are very disabled and also that those with ≥ 3HDs per month should be screened for anxiety.

## Introduction

Migraine patients are usually classified into two fundamental categories: episodic (EM) and chronic migraine (CM). The established number of headache days (HDs) per month for differentiating episodic and CM is 15 days, according to the Third International Classification of Headache Disorders (ICHD-3)^1^. This division also implies differences between the two groups in disability, quality of life, the risk of comorbid diseases and the response to certain treatments^2, 3^. However, the cut-off point of 15 monthly HDs was defined based on expert consensus, with recent studies showing that there is a substantial overlap in levels of burden, anxiety and depression between patients with frequent EM, and those with CM^4–6^. Thus, Torres-Ferrús, et al.^4^ reported that patients with 10–14 monthly HDs are as disabled as those with CM. In the same vein, Chalmer et al.^5^ studied a Danish-Russian cohort and observed that patients with migraine on 8–14 HDs per month are as disabled as those with ICHD-3 defined CM (15 or more HDs per month). They proposed that the cut-off point to distinguish EM and CM should be ≥ 8 HDs per month. Furthermore, recent observations from the Chronic Migraine Epidemiology and Outcomes (CaMEO) study indicate a substantial overlap in the measures of burden and depression among respondents with 8–14 HDs per month and CM^6^. Moreover, Silberstein et al.^7^ have demonstrated that the number of HDs per month was correlated with a higher healthcare resource utilization and migraine burden.

Migraine is often associated with symptoms of depression and anxiety^8–11^, and headache frequency has been linked to a higher risk of anxiety and depression^12–14^. In addition, the presence of anxiety or depression increase the burden on individuals who experience migraine^15, 16^. The clinician caring for patients with migraine should be able to identify those who are most disabled or at high risk of depression or anxiety. It can be hypothesized that there may be a number of HDs per month after which disability and quality of life clearly worsen or the risk of depression and anxiety is especially high. Nevertheless, the studies carried out to date do not allow us to establish the headache day threshold after which the burden of migraine is greater or there is a higher risk of comorbid psychiatric diseases. Thus, the objective of this analysis of the Spanish Migraine Atlas Survey is to define the number of HDs per month after which migraine is associated with a higher risk of anxiety and depression, severe disability and lower quality of life.

## Methods

### Study design

The Atlas of Migraine in Spain 2018 was an initiative of the Spanish Association of Migraine and Headache (AEMICE), carried out by the Health & Territory Research (HTR) group of the University of Seville, in collaboration with the Study Group of Headaches of the Spanish Society of Neurology (GECSEN) and Novartis Spain.

### Data source (Survey)

A detailed questionnaire was prepared by the HTR research group, considering the opinion of a panel of neurologists, psychologists, research experts in headaches, and patients experiencing migraine. In order to establish and formulate the questions, a scientific literature review on headache was undertaken focusing on the following domains: diagnosis, comorbidity, healthcare utilization, pharmacological treatment, complementary therapies, employment and, social limitations. Additionally, we assessed the risk of anxiety and depression with the Hospital Anxiety and Depression Scale (HADS)^17, 18^, disability level with the Migraine Disability Assessment (MIDAS)^19^, and impact of migraine on quality of life with the Headache Needs Assessment (HANA)^20^. A more detailed description of the methodology can be found in a previous study using the same sample^21^.

An online cross-sectional survey, with this questionnaire, was performed within the framework of the Spanish Migraine Atlas. The questionnaire was disseminated through the AEMICE patient association and patients filled it out voluntarily and anonymously. This survey was performed in full accordance with the Spanish law on data protection. As this was not an interventional study, no ethics committee approval was required according to the Spanish law 14/2007, 3 July, on biomedical research (BOE 159, 4 July 2007). However, all patients agreed to their participation through informed consent, and provided consent for aggregated reporting of research findings, before completing the survey. The survey was carried out between June and September 2017, including patients from all Spanish regions, using a non-probability sampling methodology. Of a total of 2,653 patients with migraine who began the questionnaire, after the validation, screening and cleaning process, the valid sample was made up of 1,283 patients. However, as one of the objectives of the present study was to assess the impact of the number of HDs per month on quality of life, psychiatric comorbidities and disability, we only included the patients who answered all questions including HADs, MIDAS, and HANA validated scales. We finally included 468 migraine patients. All patients included had been seen by a doctor within the last year and had received a medical diagnosis of migraine. The classification between CM and EM was established using the number of HDs reported by patients.

Indirect and direct cost were calculated from the data obtained in the survey. The direct costs related to medical visits, tests, and emergency room visits were obtained from the prices published in the Official Bulletins of the 17 Spanish Autonomous Communities. Average rates for 2017 were used due to the variability of prices between the different Autonomous Communities. Direct non-health care costs (borne by the patient) were self-reported by patients. Indirect cost was calculated from the patient's labour productivity losses due to medical visits, sick leave, and hospital admission days related to migraine in the economically active population. All costs were expressed in Euros referring to the year 2017, with the exception of the unit price per normal working hour in Spain, which was last updated in 2015^22^. The pharmacological costs were acquired from the economic study carried out in Spain in 2012 by Bloudek et al.^23^. The annual increase in the consumer price index of pharmaceutical products^24^, was applied to the operating costs for 2010 used by Bloudek et al.^23^.

### Variables

The questionnaire included variables related to healthcare utilization (diagnostic tests, medical visits, emergency visits, and hospital admissions), service utilization incurred by patients (visits to private specialists, and other complementary treatments for migraine) and data relating to patient work productivity losses in the previous year. In addition, three validated self-administered scales were used: HADS, MIDAS and HANA.

Patients were divided into four categories according to the frequency of HDs. Patients with 0–4 HDs per month were classified as low-frequency episodic migraine (LFEM), those with 5–8 HDs per month entered into the category of medium-frequency episodic migraine (MFEM), and when the number of HDs per month was between 9–14 HD per month the patients were classified as high-frequency episodic migraine (HFEM). Finally, patients with ≥ 15 HD per month were included into the CM group.

HADS is an instrument designed for screening potential anxiety and depression rather than grading the severity of the anxiety and depression in the general population. The HADS questionnaire included 14 items, seven of which evaluate anxiety (HADS-A) and seven that evaluate depression (HADS-D). Each item is scored on a scale of 0–3, resulting in an overall score of 0–21 for both HADS-A and HADS-D to detect possible cases of depression and anxiety. According to the score obtained in the HADS scale it is possible to distinguish between no case: 0–7; borderline case: 8–10, and case: 11–21 for both anxiety and depression^17^. The risk of anxiety and depression in HADS is considered from value 8 onwards^18^.

Additionally, disability was measured using the MIDAS scale, a 5-item questionnaire designed to evaluate disability within the past three months^19^. A score of 0–270 is used to indicate the overall level of disability due to headaches based on the following grading system: grade I, little or no disability (score of 0–5); grade II, mild disability (score of 6–10); grade III, moderate disability (score of 11–20); and grade IV, severe disability (score of ≥ 21). The highest category is subdivided into grade IV-A, severe disability (scores of 21–40) and grade IV-B, very severe disability (scores of 41–270)^19^.

HANA is a migraine-specific quality of life instrument measuring two dimensions of the chronic impact of migraine: frequency and bothersomeness. This scale contains the following seven domains: (i) anxiety/worry; (ii) depression/discouragement; (iii) self-control; (iv) energy; (v) function/work; (vi) family/social activities; and (vii) overall impact of migraine. The HANA validation studies confirmed internal consistency and reliability^20^.

### Statistical analysis

A descriptive analysis of the population was completed through the mean and standard deviation for quantitative variables and the size and percentage for qualitative variables.

The Mann–Whitney test was used to compare the homogeneity of distribution between the number of HDs and variables with two categories. The Kruskal–Wallis test was used to compare the homogeneity of distribution between the number of HD and variable with more than two categories.

Partial correlation was used to evaluate the association between the frequency of HDs and the risk of anxiety, depression (HADS), disability (MIDAS), and quality of life (HANA) controlling by age, gender, education level, smoking, alcohol, BMI and health insurance coverage.

A scatter plot was used to represent the means of HADS anxiety, HADS depression, MIDAS and HANA versus the frequency of HD per month. To this graphical representation we added the linear trend lines for both the total and each of the established categories (LFEM, MFEM, HFEM and CM).

Predictions were made from simple linear regression equations at 95% confidence.

## Results

### Patient characteristics

The study included a sample of 468 migraine patients of whom 180 (38.5%) had CM. The mean age of patients was 36.8 (± 10.7) years and 90.2% were women. Clinical characteristics of patients with migraine are displayed in Table 1. CT scans had been performed on 31.6% of the patients and MRIs on 26.3%. Lumbar punctures had been carried out on 3.8% of the patients.

**Table 1:** Sociodemographic characteristics, Patient-reported Outcomes (HADS, HANA and MIDAS) & Healthcare Resource Utilization (N = 468, unless otherwise specified).

Variables	Mean ± SD or n (%)
**Sociodemographic**	
Age	36.8 ± 10.7
Gender. Female	422 (90.2)
Marital status. Married or in relationship; N: 466	288 (61.8)
Educational level. University; N: 467	250 (53.5)
**Lifestyle habits**	
Smoking. Yes; N: 464	99 (21.3)
Alcohol. Yes	73 (15.6)
BMI. Overweight or obesity; N: 467	166 (35.6)
**Frequency of HDs**	
LFEM (0–4 days)	108 (23.1)
MFEM (5–8 days)	100 (21.4)
HFEM (9–14 days)	80 (17.1)
CM (15–30 days)	180 (38.5)
**Healthcare utilization**	
CT-scan. Yes; N:405	129 (31.9)
Magnetic resonance imaging. Yes; N: 373	95 (25.8)
Lumbar puncture. Yes; N: 338	12 (3.6)
Number of emergency visits; N: 264	5.5 ± 11.1
Number of hospital admissions; N: 58	4.8 ± 19.7
Health Insurance. Public health; N: 467	442 (94.6)
**Diagnostic delay**	
Years; N: 362	6.9 ± 6.6
**Disease duration**	
Years; N: 466	20.6 ± 11.6
**Treatment during a migraine attack**	
Analgesic or anti-inflammatory; N: 462	378 (81.8)
Triptan; N: 462	249 (53.9)
Other; N: 462	112 (24.2)
**Preventive treatments**	
Antidepressants; N: 443	105 (23.7)
Injections of botulinum toxin (type A Botox); N: 443	60 (13.5)
Anticonvulsant medications; N: 443	54 (12.2)
High blood pressure medication; N: 443	44 (9.9)
Minerals and vitamins; N: 443	42 (9.5)
Other; N: 443	84 (19.0)
None preventive treatments; N: 443	196 (44.2)
**Mental Health**	
HADS anxiety. Risk	323 (69.0)
HADS depression. Risk	184 (39.3)
**Disability**	
MIDAS	50.3 ± 52.6
**Quality of life**	
HANA	96.5 ± 34.5
**Societal costs (in Euros)**	
Direct health care cost	3,054.25 ± 20,375.98
Direct non healthcare cost	1,600.55 ± 2,183.1
Indirect cost	5,474.62 ± 7,273.65
Total cost	10,159.43 ± 22,881.75

*PRO* patient-reported outcome; *HADS* hospital anxiety and depression scale; *HANA* headache needs assessment; *MIDAS* migraine disability assessment scale; *LFEM* (Low-frequency episodic migraine); *MFEM* (Medium-frequency episodic migraine); *HFEM* (High-frequency episodic migraine); *CM* (Chronic migraine).

In addition, the mean number of visits to the emergency services due to migraine in the previous 12 months was 5.5 (± 11.0), while the mean number of hospital admissions due to migraine in the same period was 4.8 (± 19.5). 94.6% had public health coverage (only or in combination with private).

The diagnostic delay in our sample was 6.6 years (SD = 6.6, Median = 5.0) and disease duration was 20.6 years (SD = 11.6, Median = 19.0). 81.8% of patients took analgesics or anti-inflammatory drugs during a migraine attack and 23.7% took antidepressants as preventive treatment.

The annual direct health care cost per patient was €3,054.25 (± €20,375.98), direct non-health care cost was €1,600.55 (± €2,183.1), indirect costs were calculated at €5,474.62 (± €7,273.65) and total cost was €10,159.43 (± €22,881.75) [Table 1].

According to the HADS scale, 69.0% were at risk of anxiety and 39.3% susceptible to depression. The average MIDAS score was 50.3 (± 52.6) and the average HANA score was 96.5 (± 34.5).

### Headache frequency and the risk of anxiety, depression and severe disability

With respect to mental health, those at risk of anxiety reported a higher frequency of headache (13.2 vs 9.0; p < 0.001), as did those at risk of depression (14.8 vs 9.9; p < 0.001). The HDs frequency was higher in patients with severe disability (MIDAS Grade I: 7.3 vs Grade II: 5.2 vs Grade III: 6.8 vs Grade IV-A: 10.3 vs Grade IV-B: 16.5; p < 0.001) [Table 2].

**Table 2:** Bivariate analysis between the number of HD per month and sociodemographic, life habits, healthcare utilization, mental health and disability.

Variables		Number of HD per month Mean ± SD / r correlation	P-value
**Sociodemographic **			
Age categories	16–31	12.2 ± 8.3	0.383
	32–47	10.8 ± 7.2	
	48-64	12.4 ± 8.2	
	≥ 65	13.0 ± 9.8	
Gender	Male	11.6 ± 9.4	0.490
	Female	11.6 ± 7.6	
Marital status	Single	11.3 ± 8.1	0.173
	Married/relationship	11.4 ± 7.5	
	Divorced/separated	15.7 ± 9.4	
	Widow	6.0 ± 0.0	
Educational level	No schooling	17.0 ± 12.4	0.020*
	Primary school	15.1 ± 10.0	
	Secondary school	12.4 ± 8.0	
	University	10.5 ± 7.2	
**Lifestyle habits**			
Smoking	Yes	13.4 ± 8.2	0.007*
	No	11.1 ± 7.7	
Alcohol	Yes	9.1 ± 7.2	0.002*
	No	12.1 ± 7.9	
BMI	Underweight	11.5 ± 7.7	0.541
	Normal weight	11.7 ± 7.6	
	Overweight	11.9 ± 8.3	
	Obesity	10.5 ± 8.1	
**Healthcare use**			
Scanner or CT-scan	Yes	14.3 ± 8.4	< 0.001*
	No	10.7 ± 7.3	
Magnetic resonance imaging	Yes	14.7 ± 8.5	< 0.001*
	No	10.6 ± 7.2	
Lumbar or spinal puncture	Yes	14.8 ± 8.5	0.118
	No	11.1 ± 7.6	
Health Insurance Coverage	Public	11.6 ± 8.0	0.486
	Private	13.6 ± 8.8	
	Public and private combination	11.0 ± 7.2	
**Psychological health**			
HADS Anxiety	No risk	8.9 ± 6.5	< 0.001*
	Risk	12.8 ± 8.1	
HADS Depression	No risk	9.9 ± 7.0	< 0.001*
	Risk	14.2 ± 8.4	
**Disability**			
MIDAS	Grade I	7.3 ± 7.6	< 0.001*
	Grade II	5.2 ± 3.6	
	Grade III	6.8 ± 5.9	
	Grade IV-A	10.3 ± 6.5	
	Grade IV-B	16.5 ± 7.1	
**Quality of life**			
HANA	HANA total	0.520	< 0.001*

*PRO* patient-reported outcome; *HADS* hospital anxiety and depression scale; *HANA* headache needs assessment; *MIDAS* migraine disability assessment scale; *LFEM* (Low-frequency episodic migraine); *MFEM* (Medium-frequency episodic migraine); *HFEM* (High-frequency episodic migraine); *CM* (Chronic migraine).

Those patients with higher frequencies of HDs had a higher HADS anxiety mean score (LFEM: 7.9, MFEM: 9.8, HFEM: 9.9 and CM: 11.1; p < 0.001) and HADS depression (LFEM: 4.2, MFEM: 6.3, HFEM: 6.3 and CM: 8.4; p < 0.001). The MIDAS scale increased as the frequency of HDs’ increased (LFEM: 17.7, MFEM: 34.3, HFEM: 40.3 and CM: 83.2; p < 0.001). The HANA scale increased as the frequency of HDs’ increased (LFEM: 72.1, MFEM: 88.3, HFEM: 95.4 and CM: 116.1; p < 0.001). Direct health care costs, direct non-health care costs, indirect costs and total costs were higher as the number of HD’ increased (p < 0.001) [Table 3].

**Table 3:** Bivariate analysis between the number of HD and mental health, disability and cost.

	Mean ± SD				P-value
	Number of HD				
	LFEM N: 108	MFEM N: 100	HFEM N: 80	CM N: 180	
HADS Anxiety	7.9 ± 3.4	9.8 ± 4.2	9.9 ± 3.9	11.1 ± 4.1	< 0.001*
HADS Depression	4.2 ± 3.5	6.3 ± 4.0	6.3 ± 4.1	8.4 ± 4.7	< 0.001*
MIDAS	17.7 ± 15.6	34.3 ± 31.9	40.3 ± 28.4	83.2 ± 65.1	< 0.001*
HANA	72.1 ± 31.9	88.3 ± 29.3	95.4 ± 31.1	116.1 ± 28.6	< 0.001*
DHC	888.7 ± 1,645.2	1,582.1 ± 2,676.7	1,090.7 ± 1,159.6	6,044.9 ± 32,592.1	< 0.001*
DNHC	888.9 ± 1,037.1	1,158.0 ± 1,169.1	1,496.3 ± 1,909.1	2,397.8 ± 2,894.5	< 0.001*
IC	2,106.1 ± 3,839.8	3,794.3 ± 4,957.0	6,423.9 ± 6,754.7	8,007.4 ± 8,956.1	< 0.001*
TC	3,882.4 ± 4,812.6	6,534.4 ± 6,046.4	9,010.9 ± 7,846.2	16,450.1 ± 35,117.5	< 0.001*

*LFEM* low-frequency episodic migraine; *MFEM* medium-frequency episodic migraine; *HFEM* high-frequency episodic migraine; *CM* chronic migraine; *DHC* direct health care cost; *DNHC* direct non-health care cost; *IC* indirect cost; *TC* total cost.

There was a positive linear correlation between the number of HDs and HADS anxiety (r = 0.273; p < 0.001), HADS depression (r = 0.337; p < 0.001), MIDAS scales (r = 0.519; p < 0.001) and HANA (r = 0.490; p < 0.001) and all are controlled by age, gender, education level, smoking, alcohol, BMI and health insurance coverage. Therefore, as the frequency of headaches increased, the risk of anxiety and depression increased, and the quality of life and disability of patients worsened [Table 4].

**Table 4:** Partial correlation between the number of HD and healthcare utilization, mental health and disability.

	r correlation	p-value
HADS Anxiety	0.273	< 0.001*
HADS Depression	0.337	< 0.001*
MIDAS	0.519	< 0.001*
HANA	0.490	< 0.001*

*Controlling by age, gender, education level, smoking, alcohol, BMI and health insurance coverage.

**Figure 1:**
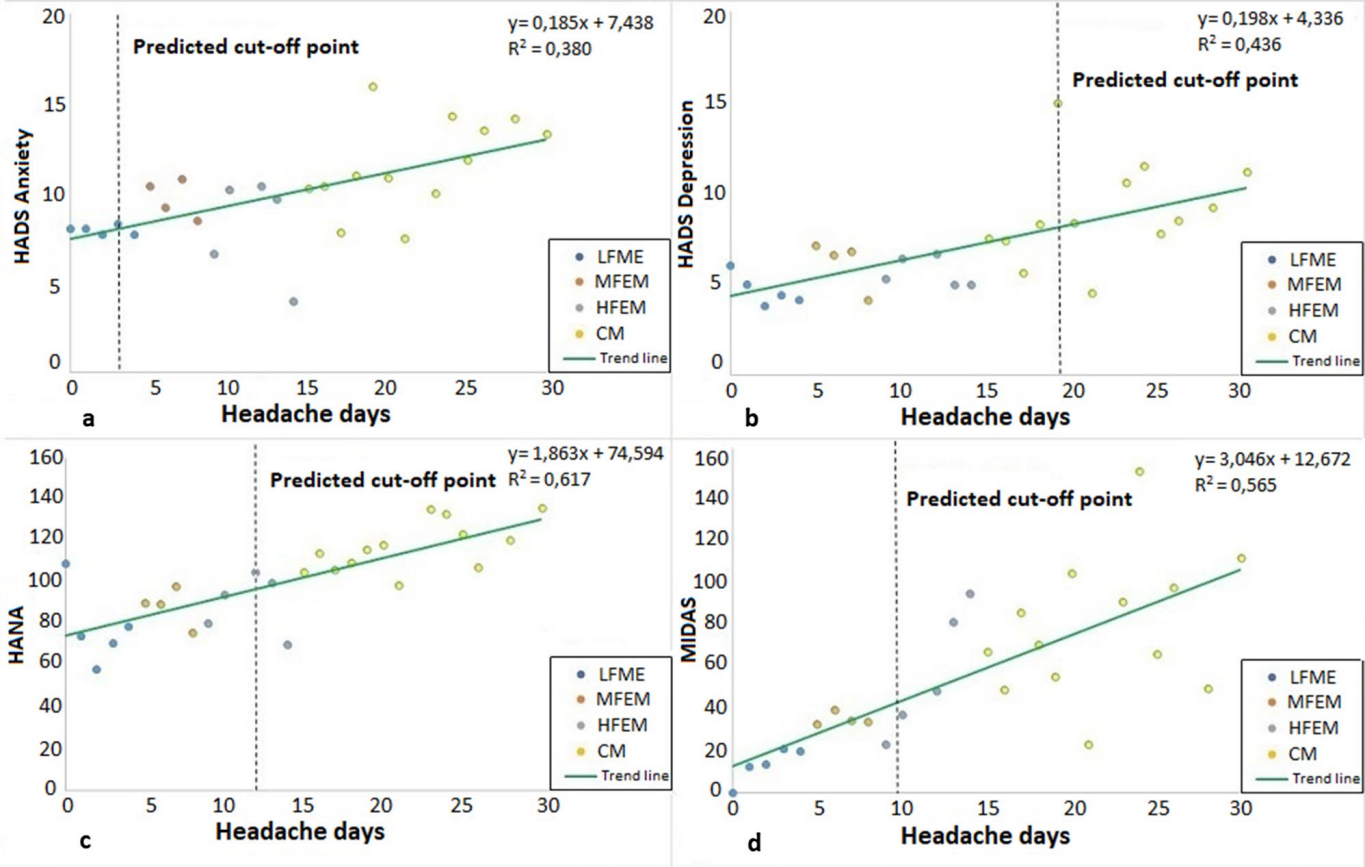
Scatter plot between anxiety, depression, quality of life, disability and number of days with headache. HADS: Hospital Anxiety and Depression Scale; HANA: Headache Needs Assessment; MIDAS: Migraine Disability Assessment Scale; LFEM (Low-frequency episodic migraine); MFEM (Medium-frequency episodic migraine); HFEM (High-frequency episodic migraine); CM (Chronic migraine).

There was a positive linear trend between the number of HDs and risk of anxiety (B = 0.185; R^2^ = 0.380; p < 0.001) [Fig. 1A], risk of depression (B = 0.198; R^2^ = 0.436; p < 0.001) [Fig. 1B], the HANA scale (B = 1.863; R^2^ = 0.617; p < 0.001) [Fig. 1C], and MIDAS scale (B = 3.046; R^2^ = 0.565; p < 0.001) [Fig. 1D].

It was found that from the third day with a headache per month (MFEM), patients became part of the population at risk of anxiety, while the risk of depression appeared from day 19 (CM). From day 12 (HFEM), the values of the HANA were higher than the mean established at 96.5. Disability (MIDAS) was severe after suffering from three or more headache/days; and became very severe after 10 or more HDs/month.

## Discussion

In the present study, the headache day threshold to identify migraine patients with severe disability is 10 HDs per month, and after 12 monthly HDs quality of life worsens above average. Our data are aligned with previous observations^4, 5, 25^ which have suggested that the headache day threshold after which migraine is associated with severe disability, lower quality of life disability, and high healthcare utilization in patients with migraine is below 15 HDs per month. Although our analysis was not predefined using a multiple regression analysis, we found a similar threshold reported by Torres-Ferrús et al.^4^ in a cohort of patients from a headache unit.

Anxiety and depression are common among migraine patients^8–11^ and impact on migraine-related disability and quality of life^26^. In our sample, we found that after three HDs per month anxiety impacts on migraine patients and interfere in everyday life. Headache frequency was previously associated with higher scores on the HADS^27^. However, anxiety may impact patients with LFEM, as observed here. Contrary to our observation, Zebenholzer et al.^15^ did not find an increased risk of anxiety in migraine patients with LFEM. Interictal anxiety is an important component of the burden of EM especially in those patients with severe migraine attacks^28^. The uncertainty of not knowing when the next migraine will occur and the severity of the attack may cause anxiety symptoms such as restlessness, persistent worrying and inability to concentrate, among others^29^. Moreover, migraine patients often endorse higher levels of neuroticism and are more likely than average to experience anxiety^30, 31^. Early recognition and management of anxiety may be of great value to improve the life of migraine patients, and, according to our findings, screening of anxiety should be considered as part of the routine clinical evaluation in patients with migraine, including those with LFEM. In this respect, the use of preventive medication should be considered when the frequency of attacks per month is two or higher, but particularly in those patients with severe disability or comorbidities such as anxiety^6, 32^.

Additionally, we studied the number of HDs after which patients have a higher risk of depression. Surprisingly, the headache day threshold from which a migraine patient has a high risk for depression is 19 monthly HDs. Different studies have shown that the risk of depression is increased in patients with frequent migraine attacks^13, 15, 33^. Furthermore, depression is a risk factor for migraine chronification^34^. In contrast with our results, previous observations suggest that migraine patients are at risk of depression when the cut-off point is somewhat below 15 HDs per month^5, 6, 25^. Multiple reasons explain why the headache threshold is so different for anxiety and depression in the present study. First, depression onset is not associated with minor episodic stressors, but to chronic stress or major life events^35^. Nineteen days per month means that patients are spending more than half their life with pain and disability, and gives weight to the consideration of depression as secondary. That realization surely will lead to hopelessness and the inability to put in place appropriate pain-coping strategies that characterize depression in migraine patients^36^. Also, the relationship between migraine and depression is complex and bidirectional^9^. Migraine may aggravate depressive symptoms and is associated with a lower improvement in mood^37^. Finally, we cannot exclude that the high headache day threshold for depression observed here is because the HADS scale only captures a significant change in those patients in whom depressive symptoms are very severe.

Among the strengths of the present study was the use of a large sample including 468 migraine patients who were evaluated using validated scales to measure anxiety, depression, quality of life and disability. However, this study is subject to several limitations. First, the diagnosis of anxiety and depression was based on the patient's response and self-reported validated questionnaires and not in-person interviews, so the clinical diagnosis cannot be verified. In addition, although HADS is widely used for detecting anxiety and depressive disorders^12^, this scale is a screening tool rather than a diagnostic test. Another limitation is that the survey was completed through an online platform, and respondents had to have access to internet, as well as knowledge of technology. In addition, a high proportion of patients were invited to participate in the survey through the association AEMICE, so patients with severe forms of migraine may be over-represented.

The increase in the number of HDs per month is associated with high disability, and a decrease in the quality of life. Anxiety may have a significant impact on migraine patients when the number of HDs is relatively low whilst depression strikes later within the headache day threshold. Our results support the need to redefine the diagnostic criteria of CM to include those patients with less than 15 monthly HDs but with high disease burden and treatment needs.

## Data Availability

The data that support the findings of this study are available from the corresponding author upon reasonable request.
